# Cardiac biomarkers and left ventricular systolic function in former very preterm infants and term controls at preschool age

**DOI:** 10.3389/fped.2024.1376360

**Published:** 2024-03-25

**Authors:** Michaela Höck, Anna Posod, Irena Odri Komazec, Elke Griesmaier, Elisabeth Ralser, Ulrike Pupp-Peglow, Ursula Kiechl-Kohlendorfer

**Affiliations:** ^1^ Department of Pediatrics II, Neonatology, Medical University of Innsbruck, Innsbruck, Austria; ^2^ Department of Pediatrics III, Division of Cardiology, Pulmonology, Allergology and Cystic Fibrosis, Medical University of Innsbruck, Innsbruck, Austria

**Keywords:** preterm infants, cardiac biomarker, N-terminal pro-B-type natriuretic peptide, high-sensitive cardiac troponin T, left ventricular systolic function

## Abstract

**Introduction:**

Due to improvements in perinatal care, survival rates of preterm infants have improved during the last decades. However, these infants remain at risk of developing cardiovascular sequelae later in life. This study aimed to investigate the cardiac biomarkers and left ventricular systolic function in former preterm infants in comparison with term controls at preschool age.

**Methods:**

The study included children aged 5–7 years old born below 32 weeks of gestational age. The control group consisted of same-age children born at term. Basic data of study participants were collected using questionnaires and follow-up databases. During the study visit, we recorded anthropometric data and blood pressure readings, determined high-sensitive cardiac troponin T (hs-cTnT) and N-terminal pro-B-type natriuretic peptide (NT-pro-BNP) concentrations, and calculated fractional shortening (FS) and left ventricular mass (LVM).

**Results:**

Term-born (*n* = 25; median gestational age, 40.1 weeks) compared with preterm-born infants (*n* = 80; median gestational age 29.6 weeks) showed no significant differences in the median concentration of hs-cTnT [median, 3.5 (IQR 3.5; 3.5) vs. 3.5 (3.5; 3.5) ng/L, *p* = 0.328] and the median concentration of NT-pro-BNP [median, 91.0 (IQR 40.8; 150.3) vs. 87.5 (50.1; 189.5) ng/L, *p* = 0.087]. FS and LVM/LVMI were not significantly different between the two groups.

**Conclusion:**

At preschool age, we observed no significant differences in cardiac biomarkers and left ventricular systolic function in preterm infants. Further studies are warranted to explore the potential of cardiac biomarkers as a prognostic tool for subclinical cardiac alterations after preterm birth.

## Introduction

Advances in neonatal care have led to an increased survival rate for preterm infants over the past decades (1). However, prematurity and low birth weight are associated with an unfavorable cardiovascular risk profile, not only in the neonatal period but also at preschool age, adolescence, and adult life (2, 3). The major determinants of cardiovascular disease are increased blood pressure, hyperglycemia, lipid alterations, and overweight/obesity, which particularly occurs after premature birth (4). Another risk factor for cardiovascular disease is altered myocardial remodeling and accelerated cardiovascular aging, which is associated with preterm birth (5). With regard to diagnosis, prognosis, and monitoring of cardiac distress, there is a strong evidence for cardiac biomarkers that provide a view into the structure and functioning of the heart (6). The most used cardiac biomarkers are troponins and natriuretic peptides. Marked elevations in cardiac troponin are sensitive biomarkers of functional and structural damage in the cardiovascular system, while a slightly increased high-sensitive cardiac troponin T (hs-cTnT) might be indicative of subclinical cardiac injury (7). N-terminal pro-B-type natriuretic peptide (NT-pro-BNP) is released by the stressed myocardium in response to volume and pressure load and is a good screening tool for detecting chronic ventricular dysfunction and heart failure (8). Elevated cardiac biomarker concentrations are associated with poor myocardial contractility and low cardiac output and correlate with echocardiographic measures of cardiac performance (9).

Prematurity entails an adverse cardiovascular risk profile, but sparse knowledge exists on cardiac biomarkers, that could identify and monitor former preterm infants with early cardiac damage already at preschool age.

This study aimed to investigate differences in cardiac biomarkers (hs-cTnT and NT-pro-BNP), left ventricular systolic function, and left ventricular mass (LVM) in former preterm infants in comparison with term controls at preschool age.

## Materials and methods

The current analysis is part of the cross-sectional study “Preterm infants and early markers for an increased risk of cardiovascular disease,” which examined former very preterm infants and term-born controls at preschool age at the Department of Paediatrics, Innsbruck University Hospital, Austria, from May 2012 to March 2015. All participants undergoing blood sampling after a minimum overnight fasting period of 8 h at the study visit were considered eligible for cardiac markers measurements. The study protocol has been published in detail previously (3). Basic data of study participants were collected using questionnaires and follow-up databases. During the study visit, we recorded anthropometric data and blood pressure readings and determined hs-cTnT and NT-pro-BNP concentrations in plasma blood samples at the Central Institute for Medical and Chemical Laboratory Diagnosis (University Hospital of Innsbruck) using an electrochemiluminescence immunoassay (ECLIA). The limit of detection of the Elecsys hs-cTnT assay (cobas e 402 and 801 analyzer) is <3 ng/L with a measuring range of 3–10.000 ng/L. The limit of blank is 2.5 ng/L, and the limit of quantification is 13 ng/L (EP17-A2, Clinical and Laboratory Standards Institute, CLSI). The limit of detection of the Elecsys pro-BNP assay (cobas e 402 and 801 analyzer) is <5 ng/L with a measuring range of 5–35.000 pg/ml. The limit of blank is 3 pg/ml and the limit of quantification is 50 pg/ml (EP17-A2, CLSI). In statistical analysis of the data, the values of cardiac markers below the limit of detection were censored by imputed values of the method detection limit divided by the square root of two (10). Pediatric-specific reference intervals and cutoffs for Roche's Elecsys hs-cTnT and NT-pro-BNP assay were published in the Canadian Laboratory Initiative on Paediatric Reference Intervals (CALIPER) cohort (11). For 1–18 years, the lower limit of hs-cTnT (90% CI) is <3 (<3, <3) ng/L, and the 99th percentile cutoff (90% CI) is 11 (11, 14) ng/L. For NT-pro-BNP the lower limit (90% CI) is <5 (<5, 7) ng/L, and the 99th percentile cutoff (90% CI) is 216 (82, 250)  ng/L (12).

A transthoracic echocardiographic examination was performed by experienced staff, using a Siemens ultrasound system equipped (ACUSON SC2000, Siemens Medical Solutions, PA, USA) with a 4- or 8-MHz transducer. Assessment of left ventricular systolic function included measurement of systolic fractioning shortening (FS), which was obtained non-invasively using M-mode echocardiography tracings. The left ventricular end-diastolic dimension (LVEDD) was measured at the R-wave of the cardiac cycle and the left ventricular end-systolic dimension (LVESD) at the end of the T-wave. FS was calculated using the following equation: FS (%) = LVEDD—LVESD / LVEDD × 100 (13). FS values of 26%–45% were defined as normal function (14). The left ventricular mass (LVM) was calculated using the Devereux's formula: LVM (g) = 0.8 × 1.04 × [(IVSD + LVIDD + LVPWD)^3^—LVIDD^3^] + 0.6 (15). The left ventricular mass index (LVMI) (g/m^2^) = LV Mass / Body Surface Area (BSA, Mosteller) (16).

Data analysis was performed using SPSS, version 29.0 for Windows (IBM Corp., Chicago, IL, USA). Descriptive statistics were used to characterize the individual variables and to determine the distribution of data. Values were expressed as numbers (frequencies, %), mean with standard deviation, and median with interquartile range. The Mann–Whitney *U*-test, Student's *t*-test, and the *χ*2 test were used where appropriate. A *p*-value *p < 0.05* was considered statistically significant.

This study was approved by the ethics committee of the Medical University of Innsbruck (Approval No. AN 4491). Verbal consent was obtained from all study participants and written informed consent from all legal guardians prior to inclusion in the study.

## Results

Cardiac biomarker measurements were available in 25 former term and 80 former preterm infants and included in this study. In the preterm group, a total number of 23 children (28.7%) were extremely preterm-born (gestational age, <28 weeks), and 57 children (71.3%) were very preterm-born (gestational age, 28–32 weeks). FS and LVM measurements were performed in 18 (72%) former term and 70 (88%) former preterm infants. With regard to sex distribution, birth weight, and maternal educational status, the study groups did not differ from respective reference populations. Gestational age in the preterm group was similar to all very preterm infants in the survey area (17). Perinatal and postnatal characteristics of all children are given in Table 1.

**Table 1:** Perinatal characteristics of the study population.

Characteristics	Term group	Preterm group	*p*-value
	(*n* = 25)	(*n* = 80)	
Maternal data			
Positive family history of CVD, *n* (%)	1 (4.0)	10 (12.5)	0.297^a^
Smoking during pregnancy, *n* (%)	3 (12.0)	23 (28.8)	0.248^a^
Maternal educational status, <12 years, *n* (%)	11 (44.0)	45 (56.3)	0.920^a^
Perinatal data			
Pre-eclampsia, *n* (%)	0 (0)	14 (17.5)	–
PPROM, *n* (%)	0 (0)	15 (18.8)	–
Perinatal asphyxia, *n* (%)	0 (0)	2 (2.5)	–
Neonatal data			
Male sex, *n* (%)	12 (48)	40 (50)	0.861^a^
Gestational age, median (IQR) (weeks)	40.1 (38.6; 40.6)	29.6 (27.6; 31.2)	<0.001^b^
Birth weight, median (IQR) (g)	3,310.0 (3,070.0; 3,775.0)	1,130.0 (960.0; 1,537.5)	<0.001^b^
Length at birth, median (IQR) (cm)	50.0 (49.3; 52.3)	38.0 (34.8; 40.0)	<0.001^c^
Small for gestational age, *n* (%)	3 (6)	8 (10)	0.776^a^

CVD, cardiovascular disease (positive family history of CVD defined as the diagnosis of congenital or coronary heart disease, angina, heart attack, or stroke in a first-degree male relative of infant and/or parent under the age of 55 or first-degree female relative of infant and/or parent under the age of 65). PPROM, preterm premature rupture of membranes. Perinatal asphyxia: ph < 7.0, BE > 16 mmol/L, 5 min Apgar < 6. SGA, small for gestational age (birth weight for gestational age and sex below 10th percentile). The values are presented as mean ± standard deviation (SD), median and interquartile range (IQR), or number (*n*) and percentage (%).

^a^ *χ*^2^ test.

^b^ Mann–Whitney *U*-test.

^c^ *t*-test.

Former preterm infants had significantly lower weight and body mass indices and significantly higher systolic blood pressure readings than their term counterparts. No significant differences in mean and diastolic blood pressure were observed. There were no significant differences in the median concentration of hs-cTnT and the median concentration of NT-pro-BNP in both groups. FS and LVM/LVMI did not significantly differ between former term and former preterm infants. Detailed findings are presented in Table 2.

**Table 2:** Characteristics at the study visit.

Variable	Term group	Preterm group	*p*-value
	(*n* = 25)	(*n* = 80)	
Characteristics at study visit			
Age at study visit, median (IQR) (year)	5.0 (5.0; 6.0)	5.0 (5.0; 6.0)	< 0.001^b^
Current weight, median (IQR) (kg)	20.3 (17.0; 22.6)	18.6 (16.8; 20.0)	0.002^b^
Current height, mean (SD) (cm)	117.0 (6.3)	114.4 (5.8)	0.059^c^
Current BMI, median (IQR) (kg/m^2^)	14.4 (13.3; 15.9)	14.1 (13.7; 15.0)	0.005^b^
Regular medication, *n* (%)	1 (4.0)	2 (2.5)	0.702^a^
Blood pressure readings			
Systolic, mean (SD) (mmHg)	98.2 (1.8)	102.3 (7.0)	<0.001^c^
Mean, mean (SD) (mmHg)	73.6 (7.6)	69.8 (7.0)	0.072^c^
Diastolic, mean (SD) (mmHg)	53.3 (6.3)	55.6 (7.1)	0.675^c^
Cardiac biomarkers			
hs-cTnT, median (IQR) (ng/L)	3.5 (3.5; 3.5)	3.5 (3.5; 3.5)	0.328^b^
NT-pro-BNP, median (IQR) (ng/L)	91.0 (40.8; 150.3)	87.5 (50.1; 189.5)	0.087^b^
Echocardiography			
FS, median (IQR) (%)	35.0 (32.0; 38.3)*	35.0 (33.0; 37.3)**	0.681^b^
LVM (IQR) (g)	43.0 (39.5; 52.8)*	42 (34.0; 53.0)**	0.210^b^
LVMI (IQR) (g/m^2^)	57.0 (50.5; 66.8)*	54 (47.0; 64.3)**	0.379^b^

BMI, body mass index (calculated as weight in kilograms divided by height in meters squared). hs-cTnT, high-sensitive cardiac troponin T. NT-pro-BNP, N-terminal pro-B-type natriuretic peptide. FS, fractioning shortening [FS (%) = LVEDD—LVESD/LVEDD × 100]. LVM (g) = 0.8 × 1.04 × [(IVSD + LVIDD + LVPWD)^3^ − LVIDD^3^] + 0.6. LVMI (g/m^2^) = LV Mass/Body Surface Area (BSA, Mosteller). The values are presented as mean ± standard deviation (SD), median and interquartile range (IQR), or number (*n*) and percentage (%).

^a^ *χ*^2^ test.

^b^ Mann–Whitney *U*-test.

^c^ *t*-test.

* *n* = 18.

** *n* = 70.

No sex-specific differences were detected for blood pressure readings: (50 females, 47 males) systolic (101.3 vs. 101.1, *p = 0.925*), mean (70.8 vs. 71.5, *p = 0.675*), and diastolic (55.8 vs. 55.7, *p = 0.980*); cardiac biomarkers: (53 females, 52 males) hs-cTnT (3.5 vs. 3.5, *p = 0.479*), and NT-pro-BNP (110.5 vs. 77.0, *p = 0.452*); and echocardiographic findings: (46 females, 42 males) FS (35.0. vs. 36.0, *p = 0.158*), IVM (42.0 vs. 42.0, *p = 0.575*), and IVMI (53.0 vs. 56.0, *p = 0.430*) in the complete study population.

In the current study, only one former preterm-born child had an elevated hs-cTnT absolute value of 12.1 ng/L and thus above the normal range (99th percentile cutoff = 11), 16 children showed an elevated NT-pro-BNP above the normal range [99th percentile cutoff (90% CI) = 216], and the highest level detected was 797.0 ng/L. Fifteen out of the children with elevated values were former preterm infants (*p = 0.073*).

There were no significant differences between children with or without elevated NT-pro-BNP in this cohort, with regard to a positive family history of cardiovascular disease, maternal educational status, smoking during pregnancy, preterm complications (chronic lung disease, brain injury, necrotizing enterocolitis, sepsis, persistent ductus arteriosus or persistent pulmonary hypertension), or risk factors for alterations in cardiac function such as pre-eclampsia, pPROM, and asphyxia. Children with minor cardiac anomalies such as patent foramen ovale (*n* = 13), atrial septal aneurysm (*n* = 1), and dysplastic aortic valve (*n* = 2) and children after ductal ligation in the neonatal period (*n* = 3) showed significantly higher NT-pro-BNP levels (*p* = 0.045).

Concentrations of hs-cTnT were within a normal range (*p* = 0.396), and FS and LVM/LVMI showed normal values and no significant difference between children with or without elevated NT-pro-BNP levels. Findings are presented in Supplementary Table S1.

## Discussion

Former preterm infants have an unfavorable cardiovascular risk profile already at preschool age with higher systolic and diastolic blood pressure readings, elevated glucose and cholesterol levels, and decreased elastic properties of the descending abdominal aorta (3, 18, 19). Recently, an association between very preterm birth and decreased markers of platelet activation among preschoolers was reported, which can also predispose to cardiovascular events (20).

With regard to diagnosis, prognosis, and monitoring of cardiac distress in former preterm infants, there is a need for an additional tool, which provides insight into the structure and functioning of the heart. Thus, we investigated whether preterm birth is associated with altered cardiac biomarkers and left ventricular systolic function at preschool age. Hs-cTnT and NT-pro-BNP are the most commonly used cardiac biomarkers in clinical care because they correspond with cardiac function, and reference values for the pediatric cohort are well established (6, 11).

Our study revealed no significant difference in the concentrations of hs-cTnT and NT-pro-BNP between former term and preterm infants. Only one patient had an elevated hs-cTnT absolute value of 12.1 ng/L and thus slightly above the normal range—a male child born small for gestational age at 31 weeks with mild respiratory distress syndrome. His NT-pro-BNP concentration (55.0 ng/L) and his FS (33%) were in a normal range, therefore clinically not significant. In the general population, a minimally increased concentration of hs-cTnT, especially when approaching the lower detection limit, may be indicative of subclinical cardiac injury (7). This could be of importance if this alteration can be detected over a longer period of time. Of the study cohort, 16 patients showed an elevated NT-pro-BNP above the normal range. Six children out of this group (37%) had minor cardiac anomalies such as a patent foramen ovale, an atrial septal aneurysm, a dysplastic aortic valve, or a ductal ligation in the neonatal period. The highest values were found in two patients: 797 ng/L in a male former preterm with 31 weeks of gestational age and mild respiratory distress syndrome and 789 ng/L in a female former preterm with 30 weeks of gestational age, who was small for gestational age and had mild respiratory distress syndrome. Their hs-cTnT concentrations were <5 ng/L and FS of 35% and 37%, respectively, again all within the normal range. Of the 16 patients with elevated NT-pro-BNP levels in our study, 15 patients were born preterm. Preterm infants are prone to different insults such as sepsis or inflammation, hypotension, or oxidant injury, which can also affect myocardial cells and result in elevated cardiac biomarkers (21). It is also known that asphyxiated neonates and infants with respiratory distress syndrome have higher levels of cardiac biomarkers (22), low cardiac output, and poor myocardial contractility (23). We could not find a significant difference between children with and without elevated NT-pro-BNP levels with regard to perinatal asphyxia, sepsis, chronic lung disease, or other known complications of preterm infants at preschool age. The reason for this outcome might be that the elevation of cardiac biomarkers occurs immediately after the damaging event and normalizes after cardiac recovery.

Previous studies reported sex-specific differences in cardiac biomarkers in childhood and adolescence (1–18 years) with higher concentrations in males (12). However, we found no significant differences between males and females for hs-cTnT or NT-pro-BNP. This might be explained by the fact that our study focused on preschool-age children. Sipola-Leppänen et al. (24) observed sex-specific differences in cardiovascular risk profiles later in adolescents. Thus, it is possible that sex-specific differences in cardiac biomarkers also become noticeable in our population after the onset of puberty, as it is already known that circulating levels of cardiac natriuretic peptides are higher in fertile women and conversely men have on average higher circulating levels of cardiac troponin (25). In contrast to the strong evidence for cardiac biomarkers in adult populations with regard to diagnosis, prognosis, and monitoring of cardiac distress, their clinical value in pediatrics is not well defined. Until now it is unclear if a slight increase in these cardiac biomarkers observed might reflect a subclinical cardiac damage already in childhood. Hs-cTnT and NT-pro-BNP are used to identify subclinical cardiac damage in adults, and those with elevated levels appear to have an associated decrease in left ventricular function, determined by echocardiography (26). Previous studies have shown a reduction in myocardial function including altered ventricular structures, left ventricular hypertrophy, and reduced systolic and diastolic function in former very preterm infants later in life, independently of elevated cardiac biomarkers (5, 27). To complete our investigation, we performed echocardiography to see if we could detect a decrease in left ventricular systolic function or left ventricular mass. Well-established normal values for FS exist for infants and children born term, that's why we focused on former preterm-born children (13). A unique cardiac phenotype characterized by smaller left ventricles with altered systolic and diastolic functions of former preterm compared to same-age children born at term at 6 years of age has been reported previously (28, 29). We used conventional echocardiography to assess left ventricular systolic function in preterm infants and compared the results with term controls and found no significant difference, which is in line with a recent study, with no significant differences in left ventricular structure and function at preschool age either (30). These findings indicate that myocardial function indices showed no sustained difference between preterm and term-born infants and a normalized cardiac function after postnatal maturation (31). The echocardiographic measurement of LVM is important in the stratification of cardiovascular risk and could be predictive of outcomes. Concordant with systolic blood pressure readings, which showed only prehypertensive values, although significantly different in the two groups, the findings in left ventricular mass index were not altered in our study group yet. Even if our analysis did not find differences between blood-based cardiac biomarkers, left ventricular systolic function, and LVM/LVMI of preterm- and term-born children, it might be a good prognostic tool of subclinical cardiac alterations for a patient group, which is steadily increasing.

## Limitations

The main limitation of our study pertains to the small sample size, which was mostly due to the difficulty of obtaining blood samples from healthy children. Thus, clinically relevant findings could have been missed because of limited statistical power. Another limitation is that we used FS to assess left ventricular function; however, FS is dependent on preload and afterload, as well as contractility. Therefore, contractility may not be accurately described by FS (32). To evaluate ventricular function in an underfilled heart or to detect a left ventricular diastolic dysfunction, new advanced and load-independent techniques such as tissue Doppler imaging (TDI) strain and strain rate (SR) and 3D echocardiography would be appropriate (14).

## Conclusion

In conclusion, we found no differences in blood-based cardiac biomarkers and left ventricular systolic function between former preterm- and term-born infants at preschool age. Further and larger studies are warranted focusing on cardiac biomarkers as potential prognostic tools for subclinical cardiac alteration after preterm birth.

## Data Availability

The original contributions presented in the study are included in the article/**Supplementary Material**, and further inquiries can be directed to the corresponding author.
