# Supplementary material for: Cardiac biomarkers and left ventricular systolic function in former very preterm infants and term controls at preschool age
Source: Front Pediatr. 2024 Mar 25;12:1376360. doi: 10.3389/fped.2024.1376360 (PMC10999603; doi:10.3389/fped.2024.1376360)
Supplement: Supplementary file 1 [file Table1.pdf]

Characteristic	NT-pro-BNP > 216 [ng/L] (n=16)	NT-pro-BNP ≤ 216 [ng/L] (n=89)	p-value
**Maternal data**			
Positive family history of CVD, n (%)	2 (12.5)	9 (10.1)	0.847[a]
Smoking during pregnancy, n (%)	5 (31.3)	21 (23.6)	0.621[a]
Maternal educational status, <12y, n (%)	6 (37.5)	50 (56.2)	0.104[a]
**Perinatal data**			
Pre-eclampsia, n (%)	1 (6.3)	13 (14.6)	0.279[a]
pPROM, n (%)	3 (18.8)	12 (13.5)	0.928[a]
Perinatal asphyxia, n (%)	1 (6.3)	1 (1.1)	0.252[a]
**Neonatal data**			
Male sex, n (%)	8 (50.0)	44 (49.4)	0.967[a]
Preterm, n (%)	15 (93.8)	65 (73.0)	0.073[a]
Gestational age, median (SD) [weeks]	30.6 (28.4; 31.4)	30.3 (28.1; 31.4)	0.524[b]
Birth weight, median (SD) [grams]	1360.0 (990.0; 1695.0)	1280.0 (1027.0; 1685.0)	0.271[b]
Length at birth, median (SD) [cm]	38.0 (35.0; 41.0)	38.0 (34.0; 41.0)	0.362[b]
Small for gestational age, n (%)	2 (12.5)	9 (10.1)	0.774[a]
**Preterm complications**			
Chronic lung disease, n (%)	3 (18.8)	15 (16.9)	0.853[a]
Brain injury (ICH 3/4, PVL, IPE), n (%)	0 (0)	2 (2.2)	0.545[a]
Necroticing enterocolitis, n (%)	1 (6.3)	3 (3.4)	0.58[a]
Sepsis n, (%)	3 (18.8)	20 (22.5)	0.74[a]
Persistent ductus arteriosus, n (%)	4 (25.0)	17 (19.1)	0.968[a]
Persistent pulmonary hypertension, n (%)	–	–	–
Minor cardiac anomaly n (%)	6 (37)	13 (14)	0.045 [a]
**Blood pressure readings**			
Systolic, mean (SD) [mmHg]	99.2 (3.6)	101.5 (7.0)	0.229[c]
Mean, mean (SD) [mmHg]	68.9 (8.1)	71.5 (7.0)	0.204[c]
Diastolic, mean (SD) [mmHg]	54.1 (5.1)	56.1 (7.4)	0.344[c]
**Cardiac biomarkers**			
hs-cTnT, median (IQR) [ng/L]	3.5 (3.5; 3.5)	3.5 (3.5; 3.5)	0.396[b]
**Echocardiography**			
FS, median (IQR) [%]	34.5 (32.0; 37.0) *	35.0 (33.0; 38.0) **	0.229[b]
LVM (IQR) [g]	39.5 (31.8; 47.3) *	42.0 (36.0; 53.0) **	0.275 [b]
LVMI (IQR) [g/m^2]	51.5 (42.3; 66.0) *	54.5 (49.0; 64.3) **	0.178 [b]

Table S1: Characteristic of children with elevated NT-pro-BNP in comparison to children with NT-pro-BNP within the normal range

Table legend

NT-pro-BNP, N-terminal-pro-B type natriuretic peptide

CVD, cardiovascular disease (positive family history of CVD defined as diagnosis of congenital or coronary heart disease, angina, heart attack or stroke in first-degree male relative of infant and / or parent under the age of 55 or first-degree female relative of infant and / or parent under the age of 65)

pPROM, preterm premature rupture of membranes

Perinatal asphyxia: ph < 7.0, BE > 16, 5 min Apgar < 6

SGA, small for gestational age (birth weight for gestational age and sex below 10th percentile, Fenton et al)

CLD, chronic lung disease/bronchopulmonary dysplasia (oxygen dependency at 36 wks PMA and/or any treatment for BPD and/or positive x-ray)

Higher degree brain injury (ICH = intracranial haemorrhage 3 or 4, PVL = periventricular leukomalacia, IPE = intraparenchymal echodensities greater than 1 cm in at least one dimension)

NEC, necrotizing enterocolitis (Bell criteria)

Sepsis (antibiotics for min. 5 days AND laboratory OR clinical abnormalities)

PDA, hemodynamically significant persistent ductus arteriosus: need of treatment - pharmacologically (Ibuprofen) or surgically (ligature)

PPHN, persistent pulmonary hypertension (based on echocardiogram and clinical findings: >10% difference between preductal and post ductal SpO2, more than two-thirds of the systemic systolic pressure)

Cardiac anomaly, patent foramen ovale (n=13), atrial septal aneurysm (n = 1), dysplastic aortic valve (n=2), ductal ligation (n = 3)

hs-cTnT, High-sensitive cardiac troponin T

FS, Fractioning shortening (FS (%) = LVEDD – LVESD / LVEDD x 100)

LVM (g) = 0.8 x 1.04 x ((IVSD + LVIDD + LVPWD)^3 – LVIDD^3) + 0.6

LVMI (g/m^2) = LV Mass / Body Surface Area (BSA, Mosteller)

[a] χ2 test

[b] Mann-Whitney- -Test

[c] t-Test

[*] n=14, [**] n=74

Values are presented as mean ± standard deviation (SD), median and interquartile range (IQR) or number (n) and percentage (%)
